# A case report of definitive endoscopic management of a large right liver lobe hydatid cyst - A novel approach

**DOI:** 10.1016/j.ijscr.2024.109825

**Published:** 2024-05-28

**Authors:** Neha Kumar, Imraan Ismail Sardiwalla

**Affiliations:** Department of General Surgery, Hepatobiliary Unit, Sefako Makgatho Health Sciences University, South Africa

**Keywords:** Hydatid disease, Liver cyst, Endoscopic, Novel, Success

## Abstract

**Introduction:**

Cystic echinococcosis is a public health concern worldwide and is endemic in rural communities in South Africa (Shaw et al., 2006). The management of hydatid liver disease is of vital socio-economic importance within the infected communities (Centers for Disease Control and Prevention [Internet]. Echinococcosis). Often, surgical intervention is needed, and this carries its own morbidity and economic burden in our low-to-middle income setting (Acta Trop., 2003). Definitive endoscopic management is rarely considered and offers an exciting option with decreased morbidity to the patient.

**Presentation of case:**

This is a case report of a 36-year-old male who presented with a large right lobe liver hydatid cyst causing abdominal discomfort and pain. He also described early satiety and weight loss with malaise. The symptoms had been present for approximately 8-months duration. The diagnosis of a hydatid liver cyst was made on positive serology and imaging (CE1). The disease was managed with medical treatment using a full course of albendazole initially and then endoscopic drainage into the duodenum using a cautery-enhanced lumen apposing metal stent. There has been no recurrence up to the present time and complete symptom and cyst resolution has been noted.

**Discussion:**

Given the success of this unconventional management, this case report will help in providing a low-morbidity management option in this endemic disease in certain selected cases. It also provides in detail how to use this option as a definitive management pathway.

**Conclusion:**

This management option required dynamic thinking and a new application of a revolutionary technology which has changed endoscopic management of a variety of conditions.

## Highlights

• Echinococcus granulosus is an endemic disease in rural South Africa as well as many other parts of the world.
• Complications are seen in up to one third of patients with hydatid liver disease
• A large, symptomatic hydatid cyst was managed definitively using endoscopic internal drainage.
• This is an innovation of the growing role of endoscopic ultrasound and cautery-enhanced lumen apposing metal stents.

## 1 Introduction

Human echinococcus is caused by the larval stages of cystodes of the genus Echinococcus [1]. There are several zoonotic genotypes within the Echinococcus granulosus complex and it is the “classical” *E. granulosus* G1 genotype that is most prevalent in the rural communities of South Africa [1]. The adult tapeworm resides in the small intestine of the definitive host (wild and domestic canids) [2]. The gravid proglottids release eggs that are passed in faeces and are immediately infectious. After ingestion by a suitable intermediate host (humans are an aberrant intermediate host), the eggs hatch in the small intestine and release oncospheres which penetrate the intestinal wall and migrate through the circulatory system into various organs especially the liver. The infection remains asymptomatic for years before the cysts grow large enough to cause symptoms in the liver [2].

Table 1 shows the World Health Organization (WHO) informal working group on Echinococcus (IGWE) classification for hydatid liver disease based on ultrasonographic features of the cyst [3]. This is a widely accepted system of classification and has consequences on treatment decision making [4].

**Table 1:** WHO classification for hydatid liver disease [4].

WHO IWGE 2001	Description	Stage
CE1	Unilocular, anechoic cyst with double line sign.	Active
CE2	Multi-septate honeycomb cyst.	
CE3a	Cyst with detached membranes.	Transitional
CE3b	Cyst with daughter cysts in solid matrix.	
CE4	Cyst with heterogenous hypoechoic/hyperechoic contents. No daughter cysts.	Inactive
CE5	Solid plus calcified wall.	

Complications are seen in one third of patients with hydatid disease of the liver [5]. These include symptoms from compression, distortion of neighboring structures or viscera, infection and erosion into the bile duct, the pleural space, or the peritoneal cavity. The diagnosis can be challenging to make and requires a combination of clinical findings, imaging, and serology [5].

In this case report, a large liver hydatid cyst was managed definitively in a novel fashion with a combination of medical therapy and followed by endoscopic internal drainage. This case report contributes to the options of management for this prevalent and challenging condition. It illustrates in detail how this management was carried out with all its nuances in the modern endoscopic unit.

## 2 Methods

The information in this case report was obtained from retrospective chart review and is reported in line with the SCARE guidelines [6].

## 3 Presentation of case

The 36-year-old patient was employed informally as a gardener in his local community. He had no pre-existing medical conditions and had no prior surgical interventions. He had no relevant travel history. He had noted weight loss and upper abdominal discomfort over the past eight months. He sought medical assistance when he noted early satiety and worsening pain.

**Fig. 1:**
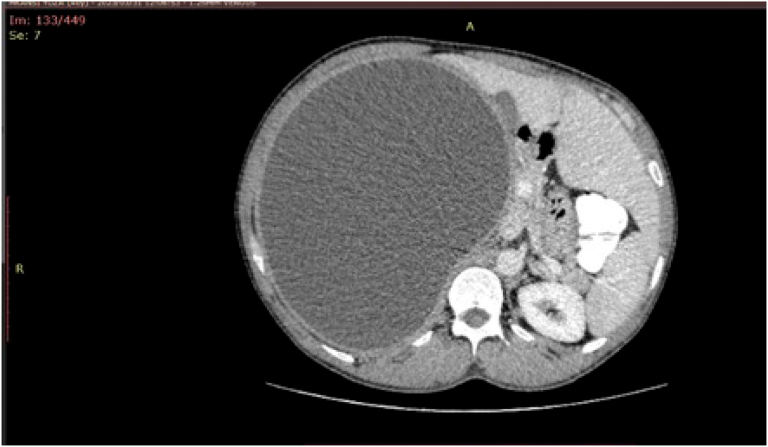
Initial CT scan of the abdomen.

His examination was notable for temporal muscle wasting and his abdominal examination revealed a diffusely tender right upper quadrant with fullness. His liver was noted to be large at 16 cm in size. No splenomegaly was noted. The remainder of his abdomen was unremarkable. The remainder of his systemic examination was also unremarkable. Ultrasound assessment of his abdomen showed a large cyst (10X13cm) with a thick wall in the liver. There were no dilated ducts within the liver and no gallstones. The remainder of the abdomen was normal. A subsequent abdominal CT scan was performed, and it showed the large liver cyst in the right lobe with no septations and a thick double wall (Fig. 1). The remainder of the liver parenchyma was normal. There was no indication of any cyst-biliary communication.

The hydatid serology was positive. Remainder of the biochemical workup including amebic serology and tumor markers were all negative. The liver function tests were also completely normal.

The patient was treated with albendazole at 15 mg/kg/day for a 28-day course and repeated for 2 cycles. The patient was re-imaged with a CT scan and the size of the cyst was unchanged. There were no changes in the liver parenchyma or biliary system. A multi-disciplinary discussion as well as discussion with the patient and his family was held. The multi-disciplinary meeting discussed the option for endoscopic drainage of the cyst and feasibility was considered as a good window between the stomach and duodenum and cyst was noted. The patient declined any surgical or percutaneous intervention and the option for endoscopic drainage was discussed and informed consent was obtained. There was a frank discussion about the novel nature of this mode of management and that worrisome consequences (intestinal perforation, peritoneal contamination, peritonitis, sepsis, need for surgery in the event of a complication, risk to life) if this should fail. The main concern around improper placement of the stent was thoroughly discussed. The patient was aware that should an inadvertent complication occur, he would likely need surgery to manage the complication. The patient was very keen to try the endoscopic option and consented accordingly.

**Figs. 2 and 3:**
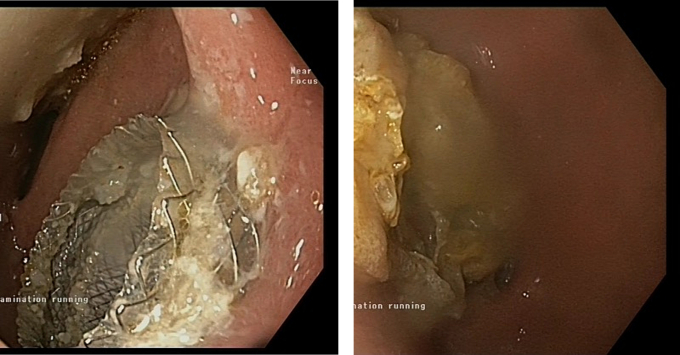
Endoscopic images of the stent insertion and debris within the cyst cavity, respectively.

The procedure was conducted in the endoscopy suite under deep conscious sedation with continuous hemodynamic monitoring. The echoendoscope was introduced through a mouthpiece and guided down to the stomach and the duodenum was intubated. A clear view of the cyst was obtained in the first part of the duodenum. A point where there were no intervening vessels was identified. A lumen apposing stent (HotAxios system 15X15mm) system was introduced with the Erbe cable attached to the stent mechanism. The stent was deployed under the sonar vision and successful deployment was confirmed as fluid from the cyst was visualized (stent can be seen in Fig. 2). A thinner pediatric scope was then also inserted through the stent into the cyst cavity and debris was visualized within the cavity (Fig. 3).

The scope was used to suction out as much fluid as possible and the patient was allowed to regain full consciousness prior to removing the scope to ensure no aspiration of the cyst contents would occur. The patient was admitted overnight, and 3 doses of prophylactic antibiotic was given as well as analgesia as required. He was also kept on a liquid diet overnight and then allowed to graduate his diet from there. He was then discharged home with a plan to follow up at the outpatient department every two weeks.

The aspirated fluid (which was straw colored in nature) was sent for analysis. The result showed no bile in its constituents and the microscopic examination did not show any active micro-organisms.

**Fig. 4:**
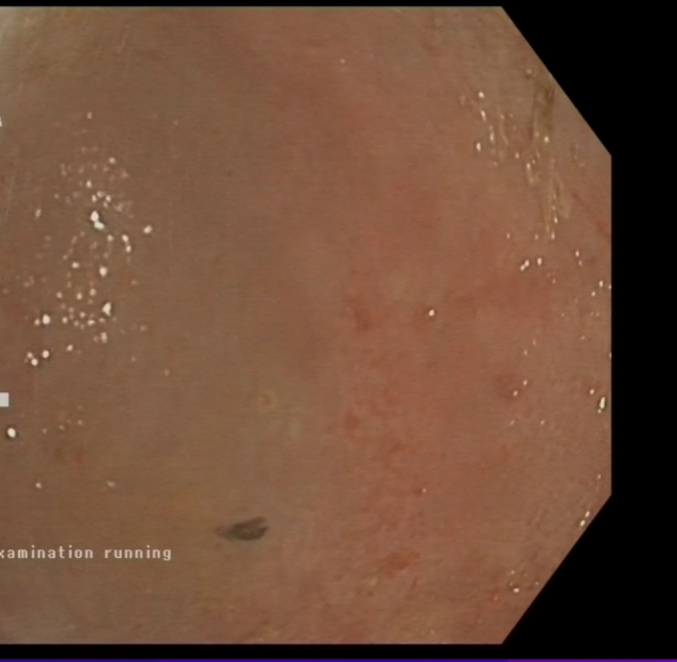
Endoscopic image of the residual cavity after stent removal.

At the follow up visits, the patient was noted to be clinically well and able to perform his activities of daily life. A repeat scope procedure was scheduled at 4 weeks post the procedure. A straight viewing scope was inserted through the stent and into the cyst cavity- the cavity was notably smaller and no longer had any fluid within it (as seen in Fig. 4).

**Fig. 5:**
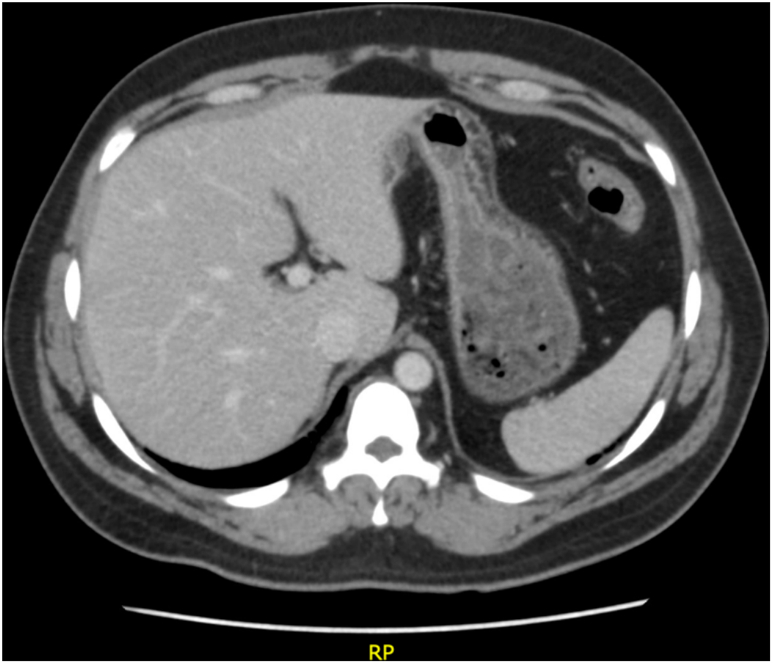
Abdominal CT scan showing complete resolution of the liver cyst.

The lumen-apposing stent was removed at this point to avoid any bleeding as an unwanted adverse event of the stent. The patient continued to follow up and had no recurrence of symptoms. A repeat CT scan showed complete disappearance of the cyst cavity and the patient remained asymptomatic (as seen in Fig. 5).

His biochemistry also remained completely normal. The patient was requested to follow up yearly after a close monitoring period of 6 months.

He has remained asymptomatic and with no recurrence on imaging for over two years now.

## 4 Discussion

Cystic echinococcosis (CE) is a worldwide zoonotic parasitic disease caused by the larval stage of Echinococcus granulosus [2]. The disease is a burden on public health in many countries, including South Africa [1]. Treatment of hydatid liver cysts can include medical therapy, percutaneous drainage, or surgical intervention (open or laparoscopic approach) [5]. Intrabiliary rupture is the most common and serious complications of hepatic hydatid cyst and is usually managed with an endoscopic retrograde cholangiopancreatography (ERCP) [7].

Surgery is still the primary management option for hydatid liver disease [8]. However, the main drawback for surgical intervention is the mortality and morbidity associated with it [8]. The post-operative mortality ranges between 2 and 7.5 % and post-operative morbidity can often exceed 60% [8]. Morbidity is specifically conferred by bile leaks, bilomas, deep suppuration, septic shock, haemobilia, fecal fistula and chest complications [8]. There are also more radical and more conservative variations in the surgical procedures and the radical procedures are considered aggressive for a benign disease process [9]. Some improvement in surgical morbidity has been obtained by the laparoscopic approach but the risk of intra-peritoneal spillage and subsequent anaphylaxis remains worrying particularly for large cysts [9]. The complications from surgery are increased when it is performed in an emergency setting especially if no medical treatment has been given beforehand [9].

The other treatment option for hydatid liver disease is the percutaneous approach. This is often done by a sonar guided or tomographic guided puncture of the cyst, aspiration of the cyst fluid, injection of a scolicidal agent (hypertonic saline, 95 % ethanol, betadine etc.) and re-aspiration of the cyst content (PAIR procedure) [10]. Adjuvant medical therapy is also often given. The PAIR procedure has been shown to be effective and less expensive when compared to surgery [11]. The main limitations of PAIR are failure in multi-vesiculated cysts, limited success in large cysts as well as a contraindication to the procedure in cases where a cyst-biliary fistula exists [12]. The main complications of PAIR are anaphylactic shock, secondary echinococcosis and chemical cholangitis [13].

There have been very few reports in literature on the evolving role of endoscopic ultrasound (EUS) in the management of hydatid hepatic cysts. These reports are largely on the use of EUS to assist in the diagnosis of intrabiliary rupture by direct visualization of the mobile hydatid membranes or cyst like material [14]. A much older series from Greece described the use of endoscopy through percutaneous drain sites into residual liver cyst cavities with the aim to inspect the residual cavity and remove any residual parasitic elements [15]. Though this study is quite old and limited in its methodology, they noted no complications of their procedure and were successfully able to remove residual parasitic elements that were delaying the collapse of the cavities as well as preventing the closure of communicated bile channels [15].

A single case study from Spain reported the accidental EUS guided drainage using a lumen-apposing metal stent of a large abdominal cyst, but this was not definitive as the patient subsequently was operated on [16]. The authors also hypothesized that this approach (even though unintended) may be a new option for patients with a massive cyst [16].

EUS guided treatment of different hepatic lesions has also been described extensively in literature [17]. With the advent of lumen-apposing stent technology, these EUS-guided interventions have become more mainstream and accepted in a variety of guidelines [17]. These interventions chiefly refer to the management of pancreatic fluid collections within the pancreatitis etiology as well as the drainage of the biliary system in advanced malignancies and inaccessible anatomy [17]. In one of the most recent systematic review and meta-analysis about EUS guided vs ERCP guided biliary drainage, the authors concluded that in malignant distal biliary obstruction, using EUS guided biliary drainage is associated with an almost non-existent risk of procedure related pancreatitis, a lower procedure time and increased ease of use in centers with expertise [18].

Concerns with endoscopic management relate primarily to the stent release process as it is crucial to place the stent correctly [19]. This requires experience and technical skill to perform correctly. Some of the complications of improper stent deployment are bleeding (including delayed bleeding), stent migration and buried stent syndrome (due to traction on the stent as the cavity collapses) [19]. These complications are infrequent (incidence of 0.59–9 %) but they carry significant morbidity if they should occur [19]. Thus, one of the key factors is to perform these procedures in high volume centers (such as ours) where the expertise is also high.

Based on these data trends as well as having the necessary expertise in our high volume, tertiary academic center, we were able to offer our patient a novel minimally invasive method of management for his large hydatid hepatic cyst. The procedure was performed after medical therapy of the Echinococcus parasite rendering it inactive. The management was successful and allowed us to avoid the morbidity that accompanies surgical intervention. The innovation of EUS and the stent technology that allows it to play a more interventional role continue to bring new options for the management of these challenging cases and these options can be considered for certain patients.

Table 2 summarizes the suggested management options that are acceptable standards for hydatid liver disease based on WHO classification of the disease [20]. Our novel approach potentially has a role in the same group of patients where PAIR is a possible treatment option.

**Table 2:** Suggested management based on WHO classification of hydatid liver disease [20].

WHO classification	Suggested management
CE 1	Albendazole alone if <5 cm. PAIR+Albendazole if >5 cm.
CE2	Surgery+Albendazole Or Non-PAIR percutaneous treatment+Albendazole
CE3a	Albendazole alone if size <5 cm. PAIR+Albendazole if >5 cm.
CE3b	Surgery+Albendazole. Non-PAIR percutaneous treatment+Albendazole.
CE4 and 5	Wait and watch.

## 5 Conclusion

Hydatid disease is endemic and common in our South African setting. The management options are multiple and more invasive options are fraught with morbidity. Endoscopic ultrasound guided interventions allow a new route and approach to the management of cystic collections in the liver. Though this needs careful selection and discussion with the patient, an EUS guided drainage of the hydatid hepatic cyst is an exciting new definitive management option.

## Consent

Written informed consent was obtained from the patient for publication of this case report and accompanying images. A copy of the written consent is available for review by the Editor-in-Chief of this journal on request.

## Ethical approval

Ethics approval was not obtained as ethics approval is not required for case reports at institute as case reports are deemed not to constitute research at our institute (SMU).

## Funding

This research did not receive any specific grant from funding agencies in the public, commercial, or not-for-profit sectors.

## Guarantor

Dr Neha Kumar and Dr Imraan Ismail Sardiwalla.

## Research registration number

1. Name of the registry: N/A
2. Unique identifying number or registration ID: N/A
3. Hyperlink to your specific registration (must be publicly accessible and will be checked): N/A.

## CRediT authorship contribution statement

Dr Neha Kumar: conceptualization, methodology, investigation, resources, data curation, writing- original draft, review and editing, visualization, project administration.
Dr Imraan Ismail Sardiwalla: conceptualization, methodology, investigation, resources, data curation, writing- review and editing, visualization, supervision, project administration.

## Declaration of competing interest

No conflicts of interest to disclose.
